# Impact of Low-Pressure Plasma Treatment of Wool Fabric for Dyeing with PEDOT: PSS

**DOI:** 10.3390/ma15144797

**Published:** 2022-07-08

**Authors:** Julija Petkevičiūtė, Audronė Sankauskaitė, Vitalija Jasulaitienė, Sandra Varnaitė-Žuravliova, Aušra Abraitienė

**Affiliations:** 1 Center for Physical Sciences and Technology, Department of Textiles Technology, Demokratu˛ Str. 53, LT-48485 Kaunas, Lithuania; audrone.sankauskaite@ftmc.lt (A.S.); sandra.varnaite.zuravliova@ftmc.lt (S.V.-Ž.); ti@ftmc.lt (A.A.); 2 Center for Physical Sciences and Technology, Department of Characterisation of Materials Structure, Saulėtekio al. 3, LT-10257 Vilnius, Lithuania; vitalija.jasulaitiene@ftmc.lt

**Keywords:** conductive textiles, plasma treatment, surface modification, PEDOT: PSS, wool fabric

## Abstract

This study presents the effect of non-thermal plasma modification on the changes of surface morphology, color characteristics and electrical conductivity of wool fabric dyed with intrinsically conductive polymer (ICP) poly (3,4-ethylenedioxythiophene) polystyrene sulfonate (PEDOT: PSS). The wool fabric was treated with an aqueous dispersion of PEDOT: PSS, Clevios F ET, providing electrically conductive properties to textiles. The wool fabric, containing basic groups of amines (NH_2_), was pre-activated with low-pressure plasma of non-polymer forming nitrogen (N_2_) gas before exhaust dyeing with PEDOT: PSS at 90 °C was applied. This treatment imparted hydrophilicity, reduced felting, increased adhesion, improved dye ability and ensured that more PEDOT: PSS negatively charged sulfonate $\left(-{\mathrm{SO}}_{3}-\right)$ counter ions would be electrostatically bounded with the cationic protonated amine groups of the wool fiber. Initially, before (N_2_) plasma treatment and after fabrics were evaluated according to the test method for aqueous liquid repellency, the surface morphology of the plasma-modified and -unmodified wool dyed fabric was observed with scanning electron microscopy (SEM). The functional groups introduced onto the surface after N_2_ gas plasma treatment of wool fabric were characterized by X-ray photoelectron and FTIR-ATR spectroscopy. The results of color difference measurements show that N_2_ gas plasma treatments provide more intense color on Clevios F ET dyed wool fabric and retain its electrical conductivity.

## 1. Introduction

Nowadays, creating wearable electrically conductive textiles is important that do not impair the usual properties—wear comfort, air permeability, flexibility, softness, resistance to repeated washing and mechanical impact. The electrical conductivity of conventional textile materials can be achieved using conductive additives of various types (metals, carbon, polymers) or conductivity enhancing finishing. Due to the broad spectrum of electrostatic properties, electrical conductivity and electromagnetic shielding as well as the relatively low cost of inherently conductive polymers (ICPs), different application scenarios in textile materials have been explored [1,2]. Due to variable electrical conductivity, electromagnetic shielding, electrostatic properties and the relatively low cost of ICPs, the potential of these materials for textile applications has been explored. PEDOT: PSS, such as ICP, is used to form conductive coatings or electrically conductive tracks, introduced into textiles by traditional finishing methods (dyeing [3], screen printing [4,5], inkjet printing [6] or coating using binders [7]) and reacts directly with the fiber’s functional groups. The resulting materials are mainly used in the development of electronic, conductive, smart and other textile materials. Conductive polymer PEDOT: PSS is lightweight, forms flexible film and can be applied onto the textile without affecting its flexibility and softness. The electrical properties of textiles coated with conductive polymers are influenced by many factors during the finishing process, such as the concentration of chemicals used, the thickness of the coating layer, the fiber content of the textile and the adhesion of the coating to the substrate [5,7]. Coatings generally do not change the flexibility of the fabric if coated with a small widespread amount of the polymer. The requirements for a perfectly conductive textile are not only sufficiently high corresponding electrical efficiency, but also constant conductive properties, namely, fastness to laundering or other wear and tear. Nevertheless, modern coating technologies do not ensure a strong enough bond of the fabric substrate to the ICP polymer. As a result, the conductivity of the coated textile is usually lost due to weak adhesion when worn or laundered. Therefore, there is a need to improve the durability of ICP-coated fabrics.

PEDOT: PSS as ICP is often chosen due to its commercial availability, stability, easy processing during film formation on various substrates and high conductivity compared to other ICPs [8] or metals [9]. This polymer system is applied to textiles to give them an electrical conductivity property. In this study, electrical conductivity was obtained for wool fabrics dyed with PEDOT: PSS aqueous dispersion Clevios F ET, (Heraeus Holding GmbH). However, PEDOT: PSS film is prone to delamination and dispersion due to the presence of a water-soluble PSS chain and this limits their use [10]. Furthermore, the fiber content of fabric, thickness of formed conductive film, interaction between fiber functional groups and polymer and other factors affect the introduction of PEDOT: PSS to the textile materials. Chemical compounds with ionizing groups present in acid dyes can be used to produce a wide range of functional finishes for textile products applied in conventional dyeing and coating processes [11]. Many acid dyes gain their acidic properties due to the presence of sulfonic acid $\left(-{\mathrm{SO}}_{3}H\right)$ or nitro $\left(-{\mathrm{NO}}_{2}-\right)$ functional groups [12]. Acidic dyes are water-soluble anionic dyes primarily used for nitrogenous fibers, such as wool, silk and polyamide, which contain basic groups [13]. Studies classify PEDOT: PSS as a “conductive acid dye”, which can bind to protein fibers due to the electrostatic interaction of the negatively charged sulfonate $\left(-{\mathrm{SO}}_{3}-\right)$ ions of the PSS chain with the cationic sites of the protein fibers [11]. The main functional groups for wool fabric are anioactive carboxy (–COOH) and cationic amino (-NH_2_) groups [11,14]. Müller and Ryan investigated how to produce wash and wear resistant conductive silk yarns dyed with conjugated polyelectrolytes [15,16,17]. The interaction of protein-based fibers with ICP, PEDOT: PSS and poly(4-(2,3-dihydrothieno[3,4-b]-[1,4]dioxin-2-yl-methoxy)-1-butanesulfonic acid (PEDOT:S) at various pHs with respect to the conductance of the protein fibers was investigated by Muhammad [18]. Synthetic polyamides are long chain polymers with repeating cationic amide (–CONH–) groups that have related characteristics and are capable of ionic bonding as protein fibers. An adhesion for polyamides by plasma treatment applied prior to glycerol-doped PEDOT: PSS polymer coating was improved in a study [19]. It has been observed that the resistivity of PEDOT: PSS-coated fibers and textiles to laundering and abrasion remains a complicated task [16,20,21].

The formed conductive polymer coating does not provide sufficiently strong adhesion between the textile and the conductive polymer layer. Due to the poor adhesion between the textile material and the polymer, the coating separates (delaminates) during wear or after washing. One method of improving the adhesion is to clean the surface of the material with a low-pressure plasma. For surface modification of heat-sensitive polymeric and textile materials, low-pressure plasma is suitable. Low-pressure plasma sources operate under vacuum, typically between 0.01 and 10 mbar, for this reason it is characterized by a high concentration of reactive species, excellent chemical selectivity and good uniformity over a large surface area [22]. During plasma processing, many electrons, free radicals, ions and metastable ions in the ion flow can break the molecular chain on the surface within a short time, increase the number of active groups and unsaturated bonds and play a specific etching effect [23]. The plasma not only changes the morphology of the substrate surface, but also binds the active sites to the surface, making the surface active for further reactions [24]. The method can also be used to incorporate functional groups into the surface of the material without altering the properties of the material [22], with the advantages of convenience and eco-friendliness, and, more importantly, of being capable of activating the surface of the substrate [25,26].

With reference to previous research, the low-pressure plasma treatment was confirmed to enhance the hydrophilicity and to improve the dyeing ability of the wool. Using a particular type of plasma can create more reactive surfaces and affect the properties of the surface without affecting the elemental composition and macroscopic mechanical properties [27,28]. Therefore, substrates can be treated with a low-temperature plasma before treatment with a conductive polymer.

The novelty of this work is that a conductive wool fabric was developed by low-pressure N_2_ plasma treatment and aqueous dispersion Clevios F ET, a commercial product of electrically conductive polymer PEDOT: PSS belonging to the group of acid dyes, according to the exhaust dyeing method at 90 °C. The dependence of color change on resistance for the plasma modified and unmodified fabrics dyed with Clevios F ET was evaluated colorimetrically after washing cycles. The aim of this paper was to determine the influence of the N_2_ plasma modification on the increase of electrical conductivity of wool fabrics by dyeing with an aqueous dispersion Clevios F ET.

## 2. Materials and Methods

### 2.1. Materials

In this research, 100% woven wool fabric was used. The technical parameters of the fabric are presented in Table 1. The pure wool fabric was purchased from JSC “Drobė” (Kaunas, Lithuania).

### 2.2. Plasma Pre-Treatment

Wool fabric samples were treated with non-polymeric N_2_ gas in a low-pressure plasma equipment—Junior Plasma System 004/123 (Europlasma, Oudenaarde, Belgium)—before dyeing to achieve a better hydrophilicity and higher absorption of PEDOT: PSS, which researchers assigned to “conductive acid dyes” [11]. Selected process parameters were as follows: N_2_ gas flow—0.01 L/min, discharge power—200 W, pressure—0.4 mbar and processing time—3 min.

Aqueous liquid repellency water/alcohol solution resistance test was used to determine a fabric’s ability to repel/absorb aqueous liquids, before and after plasma treatment [29]. Different water/alcohol composition liquids with different repellency grade numbers were used to ascertain the liquid stain resistance. The lower the aqueous solution repellency grade number (0), the worse the resistance to staining of aqueous materials—this means better hydrophilicity of the material.

### 2.3. Preparation of Polymer Solutions

For imparting conductivity properties, wool fabric after and without N_2_ low-pressure plasma modification was treated with an aqueous dispersion PEDOT: PSS, Clevios F ET (Heraeus Holding GmbH, Hanau, Germany), according to an exhaust dyeing method at 90 °C temperature. The main characteristics of Clevios F ET are presented in Table 2.

### 2.4. Dyeing with Clevios F ET

Untreated and N_2_ plasma-treated 7 × 7 cm pieces of wool fabric were dyed on a laboratory dyeing machine, Ahiba Nuance ECO-B (Datacolor, Cheung Sha Wan, Hong Kong), by immersion coating method (dyeing in exhaust) at 90 °C for 30 min in a bath based on PEDOT: PSS, Clevios F ET, according to the following recipe and process parameters: 

After dyeing with PEDOT: PSS, wool pieces were dried in a laboratory oven and evaporated, TFOS IM 350 (Roaches International, Batley, UK), at 100 °C for 3 min (see Table 3).

### 2.5. SEM Analysis

The surface morphology of the coated wool fabric was investigated with scanning electron microscopy (SEM), Quanta 200 FEG (FEI, Eindhoven, The Netherlands), at 20 keV (low vacuum): electron beam heating voltage—20.00 kV; beam spot—5.0; magnification—2000×, 6500× and 120,000×; work distance—6.0 mm; low vacuum pressure—80 Pa and large-field detector (LFD). 

### 2.6. Fourier Transform Infrared Spectroscopy with Attenuated Total Internal Reflectance (FTIR-ATR) Mode

Chemical bonding investigations of untreated and plasma-treated wool samples dyed with PEDOT: PSS aqueous dispersion, Clevios F ET, were performed using FTIR-ATR spectroscopy (MA, USA). The infrared (IR) spectra of tested samples were measured by means of FTIR Perkin Elmer Frontier (MA, USA) spectrometer in ATR reflection mode (spectrum range: 600–4000 cm^−1^, resolution: 1 cm^−1^). 

### 2.7. X-Ray Photoelectron Spectroscopy

XPS characterization of different wool samples was carried out using a Kratos AXIS Supra spectrometer (Manchester, UK) with monochromatic Al Kα (1486.6 eV) X-ray radiation powered at 225 W. The base pressure in the analysis chamber was less than 1 × 10^−8^ mbar and a low electron flood gun was used as charge neutralizer. The survey spectra for each piece of fabric were recorded at a pass energy of 160 eV with a 1 eV energy step and high-resolution spectra (pass energy −10 eV, in 0.1 eV steps) over individual element peaks. The binding energy scale was calibrated by setting the adventitious carbon peak at 284.8 eV. XPS data was converted to VAMAS format and calculated with Avantage software (Thermo Scientific, East Grinstead, UK).

### 2.8. Evaluation of Conductive PEDOT: PSS Coating Durability on Washing

Each untreated and N_2_ low-pressure plasma-treated fabric samples after dyeing at 90 °C with Clevios F ET and drying processes underwent 5 washing and drying cycles. The fabrics were washed in Scourotester Computex (Budapest, Hungary), according to the LST EN ISO 105-C06 [30] standard, method A1S: washing temperature 40 °C, duration 30 min using 4 g/L of ECE reference detergent with phosphates without optical brightener. Washed wool fabrics were line dried in ambient atmosphere.

### 2.9. Color Intensity Measurements

The color difference (ΔEcmc) of the 5 unmodified and modified Clevios F ET dyed wool samples after washing was evaluated according to LST EN ISO 105-J03 [31] standard with spectrophotometer, Datacolor Spectraflash SF450 (Datacolor AG, Rotkreuz ZG, Switzerland), wavelength range of apparatus: 360–700 nm, number of readings per sample—4 and number of measured layers of fabric—4. Prior to testing, specimens were stored under conditioning conditions: standard atmosphere—temperature (20 ± 2) °C and relative humidity (65 ± 4) % (according to LST EN ISO 139 [32]). Measurements were performed for 5 elementary samples and standard deviation was about ±4σ. 

The average of 4 readings per sample was recorded, namely, color yield (K/S), the substrate absorption function (K), the scattering function of background (S) and the reflectance (R) in the visible spectrum (400–700 nm). Other color parameters, such as L*, indicating the difference between lightness (where L* = 100) and darkness (where L* = 0); C* represents the chroma/saturation of the color; a*, the red/green coordinate, is the difference between green (−a*) and red (+a*); h, the hue angle (h sample minus h standard), is the difference in hue; and b*, the yellow/blue coordinate, is the difference between yellow (+b*) and blue (−b*). 

### 2.10. Electrical Conductivity Measurements

**Figure 1:**
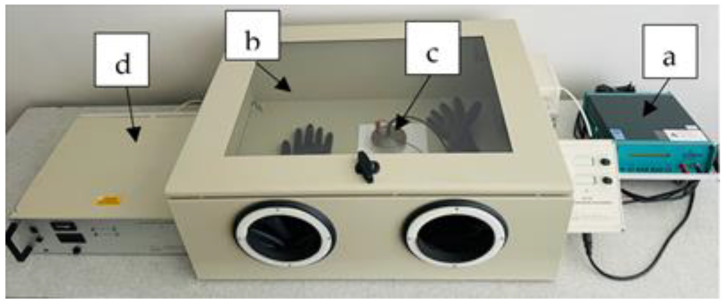
Device for surface resistance measurement: Tera-Ohm-Meter 6206 (a), JCI 191 climate control camera (b), test electrode (c) and JCI 192 dry air supply unit (d).

The electrical conductivity was measured by determining the surface resistance of woolen fabrics with a Terra-Ohm-Meter 6206 (Eltex-Electrostatik-GmbH, Weil am Rhein, Germany) according to the LST EN 1149-1 [33] standard, applying a voltage of 10 V. The range of the ohmmeter used was 10^3^–10^14^ Ω. Measurements were carried out with 5 specimens. The diameter of the electrode used—100 mm; specimens were pressed with a load of about 10 N. Measurements were carried out in dry conditions: air temperature (23 ± 1) °C and relative humidity (25 ± 5) %. Conditioning prior to testing was performed for 24 h at the same dry conditions as the measurements. The assembly of measuring devices for resistance determination is presented in Figure 1.

## 3. Results

### 3.1. SEM Analysis

**Figure 2:**
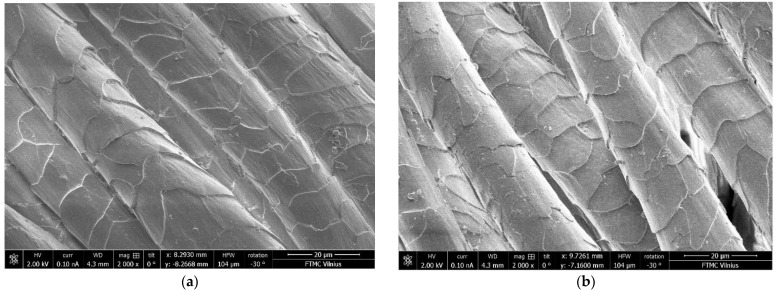
SEM images of non-treated (**a**) and N_2_ plasma-treated (**b**) wool fiber surfaces; magnification 2000×.

A scanning electron microscope can be used to investigate the morphology of the wool fiber. It can be seen from the SEM image (see Figure 2a) that the surface of the untreated wool fiber was smooth, whereas the surface of the N_2_ low-pressure plasma-treated wool fiber (see Figure 2b) was etched, resulting in a rougher surface. As excited and active plasma particles bombard the surface of the textile or polymer, they initiate a variety of reactions, such as chain scission, leading to surface etching, activation and surface cleaning [34,35]. The 120 s plasma treatment resulted in more cracks and irregularities in the surface, and such an active surface provides better penetration of dyes and chemicals. The plasma treatment removes the epicuticle layer of the wool fiber, which has a high density of cysteine bonds, is hydrophobic in nature and is a major barrier to the absorption of dyes and other chemicals [36,37]. 

**Figure 3:**
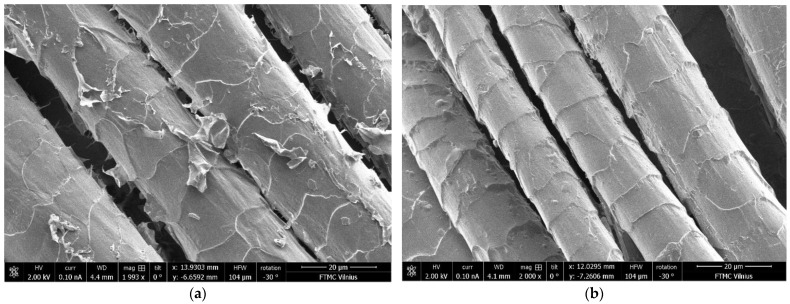
SEM images of wool fiber: Clevios F ET dyed (**a**), N_2_ plasma-treated and Clevios F ET dyed (**b**); magnification 2000×.

**Figure 4:**
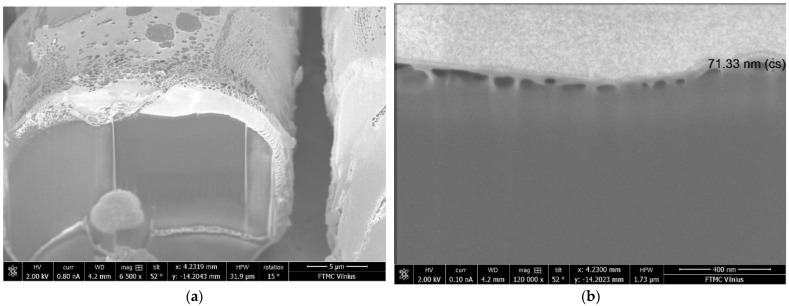
SEM images of N_2_ plasma-modified and Clevios F ET dyed wool fiber cross section; magnification 6500× (**a**) and 120,000× (**b**).

SEM images show better exhaustion and bonding of PEDOT: PSS film to the fabric samples after plasma treatment. Analysis of the SEM view of the non-treated coated substrate indicated that the thickness of the PEDOT: PSS coating is not uniform (see Figure 3a). However, the plasma-treated wool fiber surface after dyeing with PEDOT: PSS formulation Clevios F ET is more homogeneous (see Figure 3b). Conductive polymer aggregates are observed on the dyed fiber surface and the penetration depth of the conductive polymer into the fiber is about 71.33 nm (see Figure 4). The solubility of conjugated polymer PEDOT is influenced by a water-dispersible polystyrene sulphonic acid (PSS) [38,39,40].

### 3.2. Aqueous Liquid Repellency Analysis

**Figure 5:**
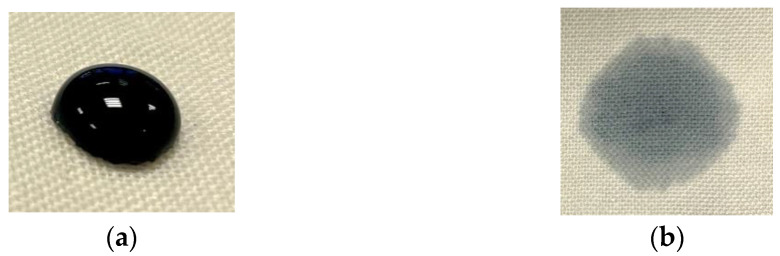
Photographs of “conductive acid dye” PEDOT: PSS and water (1:1) solution drop (see Table 3) on (**a**) untreated and (**b**) plasma-treated wool.

The increased hydrophilicity of low-pressure N_2_ plasma-gas-treated wool fabric is shown in Table 4 as a change in fabric repellency grade number. It was also found that the modified samples did not change their hydrophilicity when stored in a desiccator for 180 days. This sharp decrease in water absorption time after plasma treatment (see Figure 5b) can be explained by the increase in surface hydrophilicity and absorbency due to the formation of microcracks and the removal of scales on wool fiber surface [36,41]. The untreated sample (see Figure 5a) does not completely absorb the dye drops; it has been observed that dye drops are not absorbed, even after 30 min, thus they can be easily wiped off the surface of the fabric without getting wet. 

### 3.3. Spectrophotometric Measurements of Color Intensity

**Figure 6:**
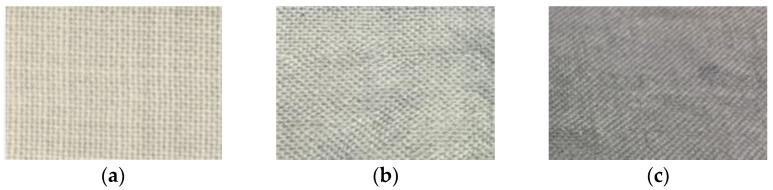
Wool fabric sample views: initial (**a**), unmodified and dyed at 90 °C with Clevios F ET (**b**) and N_2_ low-pressure plasma-modified and dyed at 90 °C with Clevios F ET (**c**).

Changes in color intensity were determined by performing color difference measurements with ColorToolsTM QC software. This method can be used to determine the ratio of lightness differences. More intense color of the material after treatment with plasma was observed (see Figure 6). It is known that non-thermal plasma processing can be used to improve the dyeing ability of various textile materials [36]. The final shade of plasma-modified and Clevios F ET in the exhaust method at 90 °C dyed wool fabric sample appears darker than that of the correspondingly dyed untreated plasma material, indicating a plasma influence on the yield of used conductive polymer (see Figure 6). Micropores in a hydrophobic epicuticular layer are treated with low-pressure plasma, which increases hydrophilicity and improves the adsorption capacity of the dyeing [42].

The measured CIE L * a * b * C * h coordinates and K/S values at the maximum wavelength of 520 nm are presented in Table 5. Although the literature sources say [43] that color yield K/S shows a higher dye ability of the material, the obtained results show that after plasma modification the value (K/S 23.45) was lower compared to the unmodified (K/S 28.56) and dyed wool fabrics. However, other values showed higher dye absorption of wool fabrics. Plasma-modified compared to unmodified and Clevios F ET dyed wool is darker in color (L * 54.47), bluer (h 177.91), more saturated colors (C 3.98) and has greener (a * −3.98) and bluer (b* 0.15) components. Plasma treatment cleans the upper greasy layer of the wool fiber, increases the best dye ability and increases the resistance during washing [36]. After 5 washing cycles, unmodified and dyed Clevios F ET wool became lighter (L 57.49), light blue (h 107.15), yellowish (b * 6.16), less green (a * −1.89), but with more saturated color (C 3.98). The untreated wool fabrics may have felted and shrunk during washing, which may have increased the color deposition, thus being the reason for the higher saturated color result [44].

### 3.4. Specific Surface Resistance Measure

**Figure 7:**
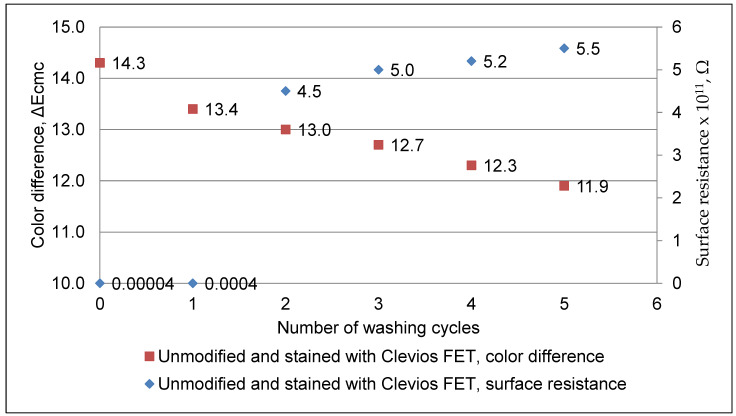
Change in color intensity after washing cycles and electrical resistance measurements of unmodified dyed with Clevios F ET wool fabric sample.

**Figure 8:**
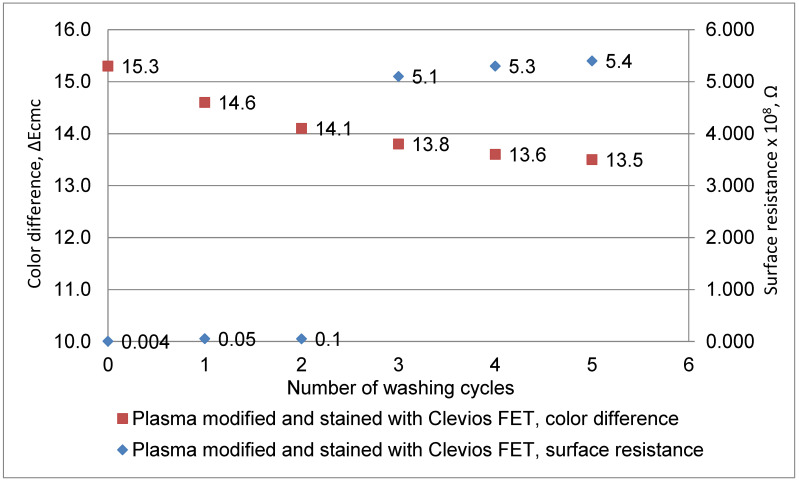
Change in color intensity after washing cycles and electrical resistance measurements of N_2_ plasma-modified and Clevios F ET dyed wool fabric sample.

Measurements of the specific surface resistance of non-treated, Clevios F ET dyed, N_2_ plasma-modified and Clevios F ET dyed wool fabric samples before and after repeated washing at 40 °C temperature were performed. The specific surface resistance of the non-treated initial sample was 5.5 × 10^13^ Ω (this value is not presented in Figure 7). The plasma-modified and Clevios F ET dyed sample demonstrated lower specific surface resistance (4 × 10^5^ Ω) (see Figure 8) compared with the unmodified Clevios F ET dyed sample (4 × 10^6^ Ω) (see Figure 7). Furthermore, the decrease in color intensity and increase of resistivity was observed after washing. Improving of color fastness to washing and dyeing intensity was observed after the plasma treatment. Furthermore, this plasma-modified and Clevios F ET dyed sample demonstrated lower specific surface resistance (5.4 × 10^8^ Ω) (see Figure 8) compared with the unmodified Clevios F ET dyed sample (5.5 × 10^11^ Ω) after 5 washes (see Figure 7). This is due to the better exhaustion and connectivity of PEDOT: PSS as “conductive acid dye” [11] to the wool fibers’ cationic amino sites after plasma treatment.

### 3.5. Fourier Transform Infrared Spectroscopy with Attenuated Total Internal Reflectance (FTIR-ATR) Mode Measurement

**Figure 9:**
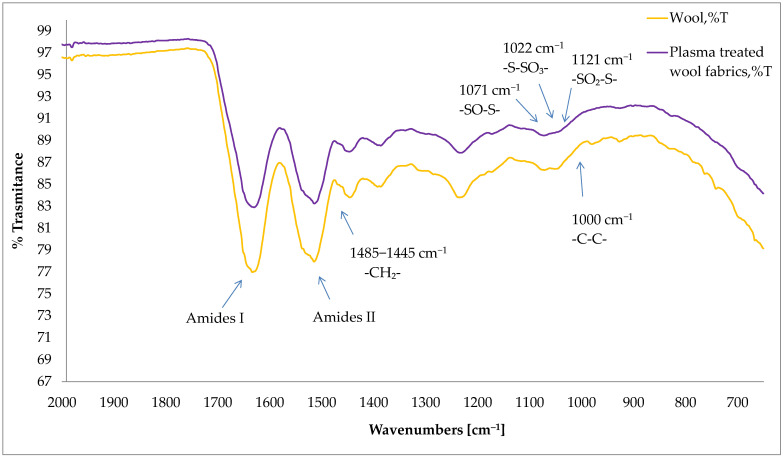
FTIR-ATR spectra illustrating untreated and plasma-treated wool fabrics.

Attenuated total reflectance (ATR) infrared spectra of the untreated and plasma-treated wool samples are shown in Figure 9. As with J. Udakhe [36], it was found that two sharp peaks in the range of 2935–2915 and 2865–2845 cm^−1^ are present in untreated fabric corresponding to methylene group’s $-{\mathrm{CH}}_{2}-$ asymmetric/symmetric stretch and correspondingly in the range of 1485–1445 cm^−1^ to the methylene (C–H) bend. Intensity of these peaks in the untreated sample is high, but after plasma modification the area of all these peaks was reduced. Both spectra show that these bands can be assigned to the amide I and amide II oscillations, which are slightly shifted at 1600 cm^−1^. They show the combination of C=O and N–H modes of amides. There is an H–O–H bending vibration at 1640 cm^−1^. There is an H–O–H bending mode at 1640 cm^−1^. M. Mori and N. Inagaki suggested that the intensity of the amide I peak should be increased after plasma treatment [36], but no significant change was detected in this study. After plasma treatment, Bunte salt $\left(-S-{\mathrm{SO}}_{3}-\right)$ formation at 1022 cm^−1^ is probably related to improved shrink-resistance properties of wool similarly detected by C.W. Kan [45]. Besides Bunte salt formation, cysteic acid was also formed as a result of the cleavage of disulphide linkage [36]. In addition to Bunte salt and cysteic acid, other interesting cystine residues formed after plasma modification, namely, cystine monoxide (–SO–S–) and cystine dioxide $\left(-{\mathrm{SO}}_{2}-S-\right)$. This indicates the oxidation of –S–S– in the surface of wool after plasma treatment [46]. Cystine monoxide and cystine dioxide are interesting because they represent a more reactive form than the parent disulphide. The cysteic acid in the polypeptide chain, together with the Bunte salt, provide a polar surface for the wool fabric, which helps to improve the wool fabric’s wettability [47]. Further, the cleavage of the disulphide bonds helps to remove the surface barrier of the wool fiber. The formation of cysteine monoxide and cystine dioxide in wool generates a more reactive substrate, which provides a suitable site for introducing agents, such as dyes and softeners, carrying nucleophilic reactive groups [48]. Furthermore, it was found that plasma treatment modified pores on the fiber surface, creating a pathway for the penetration of caustic species into the fiber during the alkali solubility test [49].

**Figure 10:**
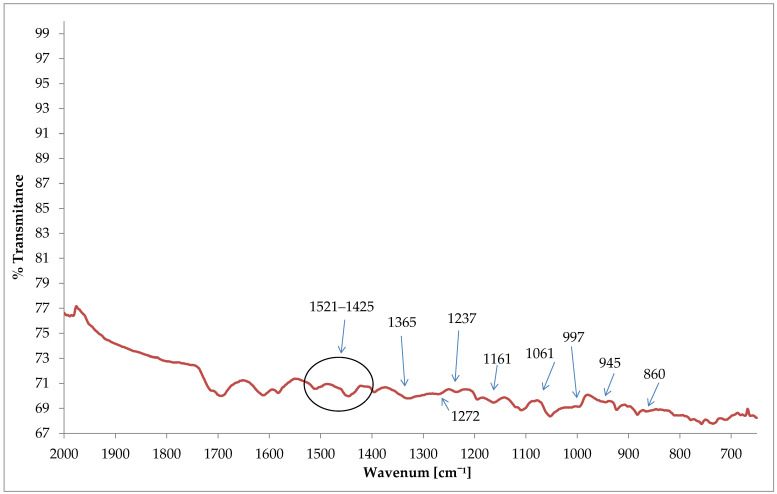
FTIR-ATR spectra illustrating the chemical composition of the Clevios FET.

ATR-FTIR spectra of chemical structure of the Clevios F ET spectra is presented in Figure 10. Similar to Hui Chung Chang and other scientists [50], the major absorption peaks were found at 1161–1170 cm^−1^ (${\mathrm{SO}}_{3}-)$ symmetric and asymmetric stretch sulfonic acid group of PSS, 1061–945 cm^−1^ (C–O–C stretching), 945–860 cm^−1^ (C–S stretching) and 1272 cm^−1^ (C=C and C–C stretching of the quinoidal structure of PEDOT). The PEDOT: PSS film showed significant peaks at around 1425 cm^−1^ and 1521 cm^−1^ due to thiophene C=C symmetric and asymmetric stretching vibrations, respectively, while the peak at 1365 cm^−1^ was attributed to C–C stretching vibrations of thiophene rings. In addition, the peaks at 1237 cm^−1^ and 997 cm^−1^ have been attributed to CH_2_ group rotation and C–O–C ring deformation [51,52].

**Figure 11:**
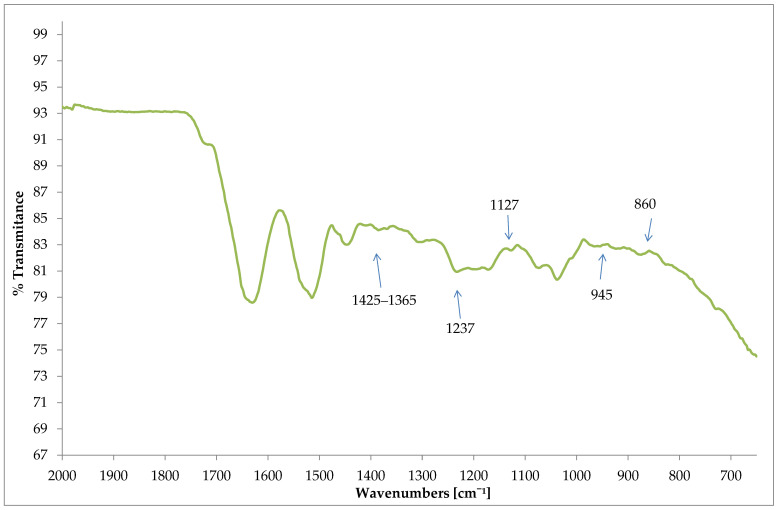
ATR-FTIR spectra of wool fabric after plasma treatment and dyeing in exhaust with PEDOT: PSS water dispersion, Clevios F ET.

ATR-FTIR spectra of wool fabric after N_2_ plasma and PEDOT PSS treatments is presented in Figure 11. All the mentioned peaks from the PEDOT: PSS dyed wool fiber were almost similar to the pristine film of PEDOT: PSS aqueous dispersion, Clevios F ET, (see Figure 10). The successful interpolation of the PEDOT: PSS dye on the wool fabric surface was evidenced by the presence of the peaks at 1425 cm^−1^ and 1365 cm^−1^, which correspond to the C = C and C-C stretching vibrations originating from the thiophene ring and increased absorption of PSS at 1127 cm^−1^ (sulfonic acid group). Mingwei Tian et al. found similar PEDOT: PSS peaks at 1517 cm^−1^ and 1300 cm^−1^ on cotton fabric surfaces [49].

### 3.6. XPS Surface Analysis

Element analysis and atomic ratio of wool treated with nitrogen plasma gas are shown in Table 6. After 120 s treatment with plasma, the micro analytical data of wool fiber had the relative atomic concentration of carbon decreased (3.22%) and the relative atomic concentration of oxygen increased (0.56%), indicating an oxidation of the adipose layer in the outer part of the epicuticle (Table 5). However, it is likely that both mechanisms contribute to the oxygen uptake. The same considerations were applied to the elemental nitrogen (N) uptake by Noeske, who also found similar evidence on the substrate surface before and after the plasma activation [53]. This increase in the (N) content of wool reflects an increase in the (-NH-) content of the wool fiber. The sulfur (S) content decreased slightly after treatment. This could be due to the fact that the treatment may have corroded the cuticle, which is full of disulfide bonds (-S-S-).

**Figure 12:**
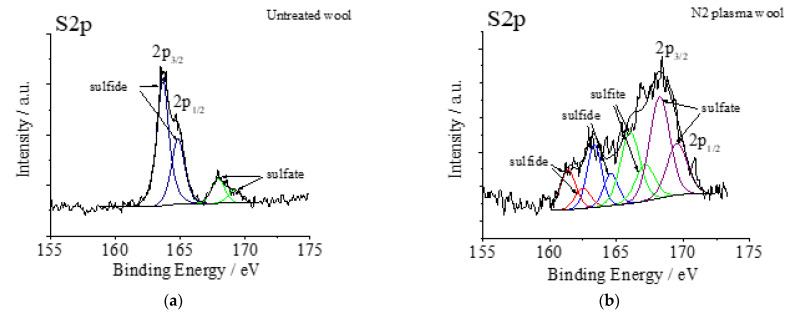
XPS spectra S2p peaks of wool fabric: untreated (**a**) and after N_2_ plasma treatment (**b**).

XPS spectra of untreated and N_2_ gas plasma-treated wool fabrics are shown in Figure 12. For the untreated wool fiber, the XPS spectrum shows two broad S2p peaks; the 163.6 eV peak intensity was stronger than the intensity at the 167.9 eV peak (see Figure 12a). After plasma treatment, the XPS spectrum intensity at the 168.2 eV peak is stronger than at the 163.3 eV peak (see Figure 12b). The increase in the oxidation states of the sulfur atoms at the fiber surface [41] is indicated by a shift of the S2p peak towards a higher binding energy. This means that the cystine residue is converted to cysteic acid. Since the 168 eV peak is quite broad, it is possible that intermediate products of cystine oxidation could have occurred [37,46,53].

**Figure 13:**
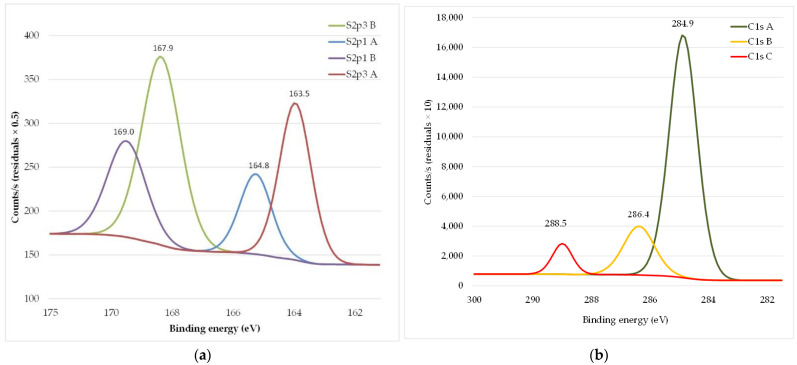
XPS spectra of the chemical composition of the PEDOT: PSS: S2p spectra (**a**) and C (1s) spectra (**b**).

Spectra illustrating the chemical structure of the PEDOT: PSS are shown in Figure 13. XPS spectral analysis revealed several characteristics of this polymer wavelength. The XPS sulfur signal indicates that the S2p peak of PEDOT: PSS is usually split into two doublets (Sp3/1A, 3/1B), representing two different chemical states of the sulfur in PEDOT: PSS, i.e., thiophene and sulfonate. The thiophene signal (from PEDOT) is detected at 163.5 and 164.8 eV, while the sulphone and sulfonate components (from PSS) are detected at binding energies of 167.9 and 169 eV (see Figure 13a) [54]. C(1s) deconvoluted into three doublet core-level spectra, a stronger peak at 284.9 eV and a shoulder at 286.4 eV, with a lower peak at 288.5 eV, which may be related to the presence of C=O bonds in some PEDOT resonant structures (see Figure 13b). The peak at 284.9 eV represents conjugated and saturated carbon atoms in the PEDOT and PSS chains. The carbon atom attached to the ${\mathrm{SO}}_{3}-$ group in PSS is only slightly displaced towards the higher coupling energy, thus its overall contribution to the spectrum is included in the 284.9 eV peak. The peak at 286.4 eV is due to the C–O–C bonds in PEDOT (Figure 13b) [55,56,57].

**Figure 14:**
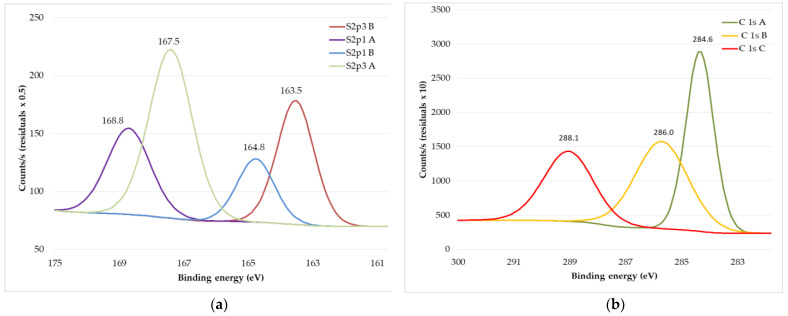
XPS spectra of wool fabric: N_2_ plasma-treated and PEDOT: PSS water dispersion Clevios F ET dyed: S2p spectra (**a**) and C (1s) spectra (**b**).

XPS spectra of previous N_2_ plasma treated and afterwards by PEDOT: PSS water dispersion Clevios F ET dyed wool fabric are presented in Figure 14. The thiophene doublet appears at 163.5 and 164.8 eV with an unsymmetrical tail that continues to the high binding energies via a positive charge on the oxidized PEDOT chain, which is partially localized in the sulfur atoms. The sulphonate ion is detected at high binding energies, around 167.5 eV (see Figure 14a). An overall increase in the relative intensity peak corresponding to PEDOT can be observed at the surface of the filament, indicating partial removal of PSS in the cup during the coating process. The small peak at 288.1 eV (C1S) could stem from the C=O bonds present in some of the resonance structures of PEDOT, which explain its increase with increasing PEDOT concentration (see Figure 14b). In addition to an increase in the intensity of the thiophene doublets, the sulfonate doublets are slightly shifted towards lower binding energies. The sulphonate signal consists of two components. One component is the sulfonate group acting as a counter ion to PEDOT^+^ SO_3_^−^ and the other is the sulfonic acid H^+^ SO_3_*^−^* group. The sulfonic acid component occurs at higher binding energies. Therefore, a shift towards lower binding energies indicates a decrease in this component with respect to the sulfonates acting as counter ions. After analysis of XPS spectra of pristine PEDOT: PSS formulation Clevios F ET, we see the same PEDOT: PSS spectra on the wool fabric, thus we can conclude that the wool fabric was coated with polymer and acquired electrically conductive properties [58].

## 4. Conclusions

It was determined that low-pressure plasma modification with N_2_ gas improved the hydrophilicity and dyeing ability of wool fibers after dyeing with conductive polymer PEDOT: PSS aqueous dispersion, Clevios FE T. Accordingly, X-Ray photoelectron spectra calculated C/N and O/C ratios suggest that the plasma modification introduces more elemental nitrogen in fiber (15.6 %), reduces the relative atomic concentration of carbon (3.22%) and an increase in the relative atomic concentration of oxygen (0.56%), suggesting the oxidation of the outermost layer of the epicuticle. FTIR-ATR spectra of low-pressure N₂ plasma-modified wool fabric samples showed a reduction of aliphatic carbon (C–C, C–H), and an increase in the content of amino groups (NH_2_), which improved the hydrophilicity of the fiber. With the formation of cystine monoxide (–SO–S–) and cystine dioxide $\left(-{\mathrm{SO}}_{2}-S-\right)$, which led to the development of a more responsive surface and the improvement of the absorption of the PEDOT: PSS polymer. For the investigated PEDOT: PSS, named as conductive acid dye, treated at 90 °C according to the exhaust dyeing method wool fabric, the interaction between fiber and PEDOT: PSS likely happens due to electrostatic interaction among protonated amino groups in the wool fiber with the negatively charged sulfonate counter ions of water-soluble conjugated polyelectrolyte PEDOT: PSS. Due to the plasma modification, a much smoother coating of the wool fiber surface with PEDOT: PSS is observed in the SEM images as well as a more intense color of the dyed fabric. After modification with plasma, wool fabric samples stained with the conductive polymer, demonstrated reduced electrical resistance during washing cycles.

## Figures and Tables

**Table 1:** Technical parameters of wool fabric.

Content of Yarn, %	Mass per Unit Area, g/m^2^	Type of Yarn: Linear Density, Tex		Weave
		Warp	Weft	Plane Wave (1:1)
Wool, 100	123 ± 3	31.0 × 2 S twist	31.0 × 1 Z twist	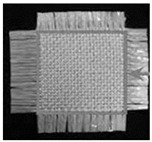

**Table 2:** Main characteristics of the Clevios F ET.

Characteristics	
Chemical composition, %	Ethane-1,2-diol ≥5–<10 Poly(3,4-ethylenedioxythiophene) polystyrene sulfonate (PEDOT: PSS) ≥1–<3 [3-(2,3-epoxypropoxy)propyl]diethoxymethylsilane ≥0.25–<1 2,4,7,9-tetramethyldec-5-yne-4,7-diol ≥0.1–<0.25
Conductivity (dried layer)	200 S/cm
Viscosity at (20 °C)	20 Pa∙s
The weight ratio of PEDOT to PSS	1:2.5
Product description (supplied form)	Aqueous dispersion

**Table 3:** Technological parameters of wool fabric dyeing with Clevios F ET.

Process	Auxiliaries	Parameters
Dyeing in exhaust	Clevios F ET, mL	500
	Deionized water, mL	500
	pH of solution	2.2
	Liquor ratio	1:20
	Temperature, °C	90
	Time, min	30
	Rotation speed of dyeing containers, rpm	20
	Temperature rise rate, °C/min	2
Drying	Temperature, °C	100
	Time, min	3

**Table 4:** Fabric repellency grade per days.

Days	1	7	30	180
Non-treated fabric, repellency grade number	3	3	3	3
Treated fabric, repellency grade number	0	0	0	0

**Table 5:** Color parameters of the experimental samples.

Sample	L *	A *	B *	C *	h	K/S (λmax = 520 nm)
Dyed with Clevios F ET	56.60 ± 0.07	−3.44 ± 0.07	1.48 ± 0.07	3.75 ± 0.07	156.79 ± 0.07	28.56 ± 0.07
Plasma-modified and dyed with Clevios F ET	54.47 ± 0.07	−3.98 ± 0.07	0.15 ± 0.07	3.98 ± 0.07	177.91 ± 0.07	23.45 ± 0.07
Dyed with Clevios F ET, after 5 washing cycles	57.49 ± 0.07	−1.89 ± 0.07	6.13 ± 0.07	6.42 ± 0.07	107.15 ± 0.07	25.36 ± 0.07
Plasma-modified and dyed with Clevios F ET, after 5 washing cycles	52.65 ± 0.07	−2.97 ± 0.07	1.66 ± 0.07	3.4 ± 0.07	150.85 ± 0.07	21.31 ± 0.07

**Table 6:** Element analysis (wt. %) and atomic ratio of wool treated with nitrogen plasma gas.

Sample	Elemental Concentration (wt. %)						Atomic Ratio	
	Si2p	C1s	Ca2p	N1s	O1s	S2p	C/N	O/C
Untreated	1.00	72.16	0.79	8.46	15.08	2.51	8.53	0.20
After plasma treatment	1.69	50.3	1.22	15.62	28.13	3.04	3.22	0.56
